# Social media behaviors and symptoms of anxiety and depression. A four-wave cohort study from age 10–16 years

**DOI:** 10.1016/j.chb.2023.107859

**Published:** 2023-07-01

**Authors:** Silje Steinsbekk, Jacqueline Nesi, Lars Wichstrøm

**Affiliations:** a Department of Psychology, Norwegian University of Science and Technology, Trondheim, Norway; b Department of Psychiatry & Human Behavior, Warren Alpert Medical School of Brown University, Providence, RI, USA; c Department of Child and Adolescent Psychiatry, St. Olavs University Hospital, Trondheim, Norway

**Keywords:** Social media, Depression, Anxiety, Adolescence, Within-person, Random intercept cross-lagged panel model

## Abstract

**Background:**

Concerns have been raised that social media use causes mental health problems in adolescents, but findings are mixed, and effects are typically small. The present inquiry is the first to measure diagnostically-defined symptoms of depression and anxiety, examining whether changes in social media behavior predict changes in levels of symptoms from age 10 to 16, and vice versa. We differentiate between activity related to one’s own vs. others’ social media content or pages (i.e., self-oriented: posting updates, photos vs other-oriented: liking, commenting).

**Methods:**

A birth-cohort of Norwegian children was interviewed about their social media at ages 10, 12, 14 and 16 years (*n* = 810). Symptoms of depression, social anxiety and generalized anxiety were captured by psychiatric interviews and data was analyzed using Random Intercept Cross-lagged Panel Modeling.

**Results:**

Within-person changes in self- and other oriented social media behavior were unrelated to within-person changes in symptoms of depression or anxiety two years later, and vice versa. This null finding was evident across all timepoints and for both sexes.

**Conclusions:**

The frequency of posting, liking, and commenting is unrelated to future symptoms of depression and anxiety. This is true also when gold standard measures of depression and anxiety are applied.

## 1. Introduction

Social media has been an essential component of western adolescents’ lives for over a decade. During this period, the prevalence of emotional problems in youth has increased (Keyes, Gary, O’Malley, Hamilton, & Schulenberg, 2019; Lebrun-Harris, Ghandour, Kogan, & Warren, 2022; Mojtabai, Olfson, & Han, 2016; Parodi et al., 2022; Platt, Bates, Jager, McLaughlin, & Keyes, 2021; Shorey, Ng, & Wong, 2022; Twenge, Cooper, Joiner, Duffy, & Binau, 2019). Unsurprisingly then, scholars have suggested that the rise in social media use may be responsible for increasing rates of anxiety and depression in adolescents (Keyes et al., 2019; Twenge, 2019, 2020). However, some studies report no associations between frequency of social media use and mental health (Jensen, George, Russell, & Odgers, 2019), some find social media use to be associated with good mental health (Fredrick, Nickerson, & Livingston, 2022), others with impaired mental health (Twenge, 2020), and when associations are revealed, they are typically small (Arias-de la Torre et al., 2020; Cunningham, Hudson, & Harkness, 2021; Ivie, Pettitt, Moses, & Allen, 2020; McCrae, Gettings, & Purssell, 2017; Odgers & Jensen, 2020; Seabrook, Kern, & Rickard, 2016; Shin, Juventin, Chu, Manor, & Kemps, 2022; Valkenburg, Meier, & Beyens, 2022).

A major shortcoming of existing research is that studies have conceptualized mental health problems in a variety of ways (e.g., reduced well-being, psychological distress, poor self-esteem, depressive symptoms). Because social media use may relate differently to different mental health problems (e.g., social anxiety versus overall well-being), these inconsistent findings may be due to studies not assessing the same phenomenon. Studies have also typically relied on self-reports of both social media use and mental health, thereby running the risk of inflating relations due to a common methods bias. Studies assessing more strictly defined mental health problems and measuring such problems by other means than self-report are needed.

Further, the specific ways youth use social media—their social media behaviors—may also be differently related to mental health outcomes. Although the majority of studies have focused on overall frequency or time spent on social media, not type of use, one line of research has differentiated between active and passive use, often reporting the former to be positively and the latter to be negatively associated with well-being (Verduyn et al., 2015, 2017). However, a recent review showed that most findings do not support such associations, and the authors concluded that the passive/active distinction is too coarse (Valkenburg, van Driel, & Beyens, 2022), thus essential nuances may be missed. In line with the latter view, the current study adds to existing research by examining how changes in specific social media behaviors (‘posting’ updates and photos on one’s own site; ‘liking’ and ‘commenting’ on others’ posts) forecast changes in the different types of emotional problems most frequently hypothesized to be related to social media use, namely symptoms of depression, social anxiety, and generalized anxiety. To ensure a strict conceptualization of these mental health problems, the current study measures symptoms assessed by psychiatric interviews of participants and their parents, using four biennial waves of data capturing ages 10–16 years. We thus expand on prior research by examining the preadolescent period, which has been neglected in prior research (Odgers & Jensen, 2020). Further, because most studies have used relatively small, nonrepresentative samples (Odgers & Jensen,2020), we rely on a birth-cohort sample of more than 800 participants.

### 1.1. Social media use and depression – theoretical assumptions and existing evidence

Adolescence is a period of social reorientation from the nuclear family towards friends and changing friendships in the process of identity formation (Marcia, 1980), which often involves an increase in potentially stressful interpersonal events (e.g., Cyranowski, Frank, Young, & Shear, 2000). As a result, developmental theories point to social interaction as an important etiological factor in explaining increased rates of depression during this age period, especially in girls (Hammen, 2018; Nolen-Hoeksema & Girgus, 1994). Social interaction is indeed one of the primary purposes of social media use. Accordingly, in considering potential mechanisms linking social media use and depression, it is reasonable to spotlight characteristics of online interactions. It has been suggested, for example, that social media use increases the risk for depression due to the lack of face-to face interactions available on these platforms (e.g., Barry, Sidoti, Briggs, Reiter, & Lindsey, 2017), given that the presence of such interactions are known to protect against depression (Cruwys et al., 2013; Lee, Murphy, & Andrews, 2019). The lack of physical and non-verbal cues (e.g., gestures, nuances in voice and tone) is one of several social media affordances that shape online interactions (Nesi, Choukas-Bradley, & Prinstein, 2018a; Nesi, Choukas-Bradley, & Prinstein, 2018b). It has been argued that these affordances may result in online interactions that fail to protect against mental health symptoms in the way that face-to-face interactions do; and, at worst, that they may even promote symptoms of mental illness. Face-to-face interactions may be perceived as richer, generating a stronger feeling of belongingness and closeness, which may promote mental health (Sherman, Michikyan, & Greenfield, 2013). Further, lack of physical cues may increase the risk for communication difficulties and conflicts (Amichai-Hamburger, Kingsbury, & Schneider, 2013; Kruger, Epley, Parker, & Ng, 2005), which may promote negative feelings and hamper feelings of connectedness and closeness, thus potentially impairing mental health. As indicated by the displacement theory (Kraut et al., 1998; Nie, 2001), social media use may simply leave less time for such face-to-face interaction. Thus, both the potential decline in offline interactions as well as the lower quality of online interactions as compared to offline ones, may link social media use and depression.

Further, interpersonal theories of depression state that depressed individuals may interact with others in ways that increase their risk for future depressive symptoms. For example, they may engage in reassurance-seeking or negative feedback-seeking, the tendency to actively solicit negative feedback from others to confirm one’s own negative self-concept (Hames, Hagan, & Joiner, 2013). Other affordances of social media, such as the countable social metrics and availability (Nesi et al., 2018b) might reinforce such behaviors (e.g., reassurance seeking behavior: increased posting to seek self-confirmation), potentially promoting increased levels of depression. Adolescents may also seek out individuals online with whom to co-ruminate (Battaglini, Rnic, Tracy, Jopling, & LeMoult, 2021)—an interactional style that may prolong or increase the risk for depression (Spendelow, Simonds, & Avery, 2017). Other potential pathways linking social media use to depressive symptoms include risk for cyber-bullying (e.g., Hamm et al., 2015), negative social comparison (Nesi & Prinstein, 2015) and promotion of increased body-concern and impaired body-image (Vandenbosch, Fardouly, & Tiggemann, 2022), which may forecast depression (Appel, Gerlach, & Crusius, 2016; Choukas-Bradley, Roberts, Maheux, & Nesi, 2022; Murray, Rieger, & Byrne, 2018).

In sum, several mechanisms may link social media use and depression, but a meta-analysis capturing the period from 2012 to 2020 concluded that associations between social media use and depressive symptoms in adolescence ranged from positive to negative (*r* = −.10 to *r* = 0.33) (Ivie et al., 2020). Other meta-analyses report positive, but small associations with self-reported depressive symptoms (Vahedi & Zannella, 2021), with comparable estimates when separating between time spent using social networking sites (*r* = 0.11) and intensity of use (*r* = 0.009) (Cunningham et al., 2021). To the best of our knowledge, the present inquiry is the first to examine the longitudinal impact of specific social media behaviors on future depressive symptoms assessed by gold-standard psychiatric interviews.

### 1.2. Social media use and anxiety – theoretical assumptions and existing evidence

Although some features of social media (e.g., lack of physical cues) may represent disadvantages for some individuals, they may create opportunities for others. For people with social anxiety, online social interaction may be less distressing than offline social interaction (Erliksson, Lindner, & Mortberg, 2020). A review confirms that many socially anxious individuals prefer online above offline communication, although such preference is more typically seen in adults than adolescents (Prizant-Passal, Shechner, & Aderka, 2016). Both social anxiety and generalized anxiety disorder in childhood are associated with difficulties in social interaction (McClure & Nowicki, 2001; Pickard, Rijsdijk, Happé, & Mandy, 2017; Scharfstein, Alfano, Beidel, & Wong, 2011) and the tendency to withdraw from social contact (Kingery, Erdley, Marshall, Whitaker, & Reuter, 2010). Social media may represent an alternative arena where anxious individuals can practice, and thus improve, online social skills as well as experience the benefits of social interaction (e.g., positive feedback, self-confirmations). A study of emerging adults found that self-reported social anxiety was associated with more frequently initiating online interactions and giving others positive feedback and support (Ross et al., 2021). Over time, such behavior may increase the frequency of online social interactions, which can enhance online social confidence and skills. Although social skills are context-specific (McFall 1982), offline and online social skills are correlated (Mantzouranis, Baudat, & Zimmermann, 2019; Resnik & Bellmore, 2019), thus improved online social skills may generalize to offline skills. The resulting increased mastery of real-life social encounters implies that repeated exposures to feared social stimuli online may *reduce* anxiety symptoms with social origins.

On the other hand, social media use may also *increase* future symptoms of anxiety. Although adolescents may initially turn to social media for emotional support, prior work suggests that social media use can lead to co-rumination in early adolescence, which forecasts more anxiety symptoms over time (Ohannessian, Fagle, & Salafia, 2021). Additionally, cognitive models of social anxiety hold that anxious individual’s self-focused attention increases negative self-evaluations which fuel anxiety (Haller, Kadosh, Scerif, & Lau, 2015). The self-exposure characterizing posting photos and updates may strengthen such self-focused attention, as may liking and commenting on other’s posts (i.e., through social comparisons), thus theoretically, these social media behaviors may increase anxiety symptoms. Further, appearance comparisons, which youth are likely to experience when using social media (Jarman, Marques, McLean, Slater, & Paxton, 2021), are found to increase social anxiety over time (Rapee et al., 2022). And, according to the displacement hypothesis (Kraut et al., 1998), being online at the expense of meeting people offline, may deprive adolescents of the needed practise of offline social skills, thus increasing their fear of failing in offline interaction — a core symptom of social anxiety. Moreover, predominantly meeting peers online may imply avoidance of feared offline social situations, and thus negatively reinforce socially anxious (i.e., avoidant) behavior.

As previously noted, most studies of social media use have focused on depressive symptoms (Sarmiento et al., 2020), and the few studies, particularly longitudinal ones, on anxiety provide inconclusive findings. Some report no direct relation between frequency of social media use and self-reported anxiety symptoms (e.g., Ohannessian et al., 2021), others find that social media use is alternatively associated with more or less anxiety (Sarmiento et al., 2020). This research has been based on self-reported anxiousness (Keles, McCrae, & Grealish, 2020), which only modestly correspond with diagnostically-defined symptoms captured by gold-standard psychiatric interviews (Sveen, Berg-Nielsen, Lydersen, & Wichstrom, 2013). Furthermore, like research on social media use and depression, most existing studies on anxiety fail to examine specific social media behaviors that may play a role. We therefore extend existing knowledge by examining the relation between social media behavior and interview-assessed, diagnostically-defined symptoms of social anxiety and generalized anxiety.

### 1.3. Do depression and anxiety forecast altered social media use?

The affordances of social media may make it more likely for anxious youth to use social media, and the same may apply to individuals with depressive symptoms. Depression is characterized by loss of interest and energy, often accompanied by reduced social contact (American Psychiatric Association, 2013), and social media may represent an alternative arena for youth with depressive symptoms to stay in touch with peers. On the other hand, because online social interaction usually takes place within the youth’s offline social network (Valkenburg & Peter, 2007), and adolescents with anxiety and depression are likely to withdraw from social contact (Hards et al., 2022), social media use may also decline as a result of such emotional difficulties. These associations may depend on the type of social media behavior, though. For example, because socially anxious adolescents favour online over offline communication for self-disclosure (Valkenburg & Peter, 2007), one might expect that increased levels of anxiety predict more social media posting. On the other hand, because public posting may generate a fear of judgement and negative feedback, increased anxiety may also predict *less* posting over time, whereas more ‘safe’ social media use, such as liking or simply scrolling, may increase.

One recent review reported that among the four identified longitudinal studies examining paths from depressive symptoms to social media use, research finds depressive symptoms to predict both more and less future social media use (Sarmiento et al., 2020). The same review identified five correlational studies of anxiety where different aspects of social media use were conceptualized as the outcome (e.g., time spent, problematic Internet use, talking online to friends), all finding anxious adolescents to display more of the social media use captured (Sarmiento et al., 2020). We extend this existing research by examining whether relations between anxiety and depressive symptoms and future social media use are replicated when using a longitudinal design. We also aim to bring clarity to previously mixed findings by assessing diagnostically-defined symptoms of depression and anxiety, rather than self-reported symptoms, and to examine type of social media use, rather than overall time spent.

### 1.4. Sex/gender differences in the relation between social media use and mental health

Females are more likely than males to display depressive symptoms, and this sex/gender^1^ difference is apparent by age 12 (Hyde & Mezulis, 2020) with girls being at least twice as likely as boys to develop depression in adolescence (Salk, Hyde, & Abramson, 2017).

The increase in social anxiety and generalized anxiety during adolescence is also more typically seen in girls than boys (Steinsbekk, Ranum, & Wichstrom, 2021). Sex/gender differences have also been revealed in terms of *how* girls and boys use social media (e.g., girls using more photo-based platforms (Lenhart, 2015); in terms of which social media behaviors predicts which *outcomes* (e.g., active Facebook-use associated with depressive symptoms in boys, but not girls, whereas the opposite sex/gender difference was found for passive use (Frison & Eggermont, 2016); and in terms of which *underlying mechanisms* link social media use and mental health problems (e.g., females being more likely to use social media for social comparisons reasons (Haferkamp, Eimler, Papadakis, & Kruck, 2012) and be more vulnerable to body image concerns, which may lead to depressive symptoms (Choukas--Bradley et al., 2022)). However, although some studies find that social media use is more strongly related to depression and anxiety in girls than boys, other do not find such differences (Arias-de la Torre et al., 2020; Keles et al., 2020; Sewall, Goldstein, Wright, & Rosen, 2022). Among studies examining the impact of mental health on social media use, one study found that girls displaying internalizing symptoms were more likely to post content characterized by negative affect and somatic complaints, whereas boys did not (Ehrenreich & Underwood, 2016). It has also been reported that for boys only, depressive symptoms are prospectively associated with increases in social comparison and feedback seeking on social media (Nesi, Miller, & Prinstein, 2017). As concluded in a recent umbrella review, the heterogeneity in methodology and inconsistency in results calls for prospective research, especially longitudinal cohort studies (Arias-de la Torre et al., 2020), such as the current inquiry.

### 1.5. Self - and other -oriented social media use and symptoms of depression and anxiety

In the present work we differentiate between posting one’s own updates and photos, termed *self-oriented social media use*, versus liking or commenting on other people’s posts, termed *other-oriented social media use*. We apply the terms self-vs. other-oriented to differentiate between activity related to one’s own vs. others’ social media content or pages. This aligns with related distinctions, such as conceptualizations of self-versus other-oriented content in theories of online self-presentation (Hollenbaugh, 2021). Although self-exposure is inherent in both self- and other-oriented social media behavior, we theorize that the former contains more self-exposure, and thus causes less upward social comparison than the latter. As a result, we suggest that these behaviors may differentially impact mental health. For example, self-presentation on social media has been found to be associated with higher self-esteem (Meeus, Beullens, & Eggermont, 2019) and posting usually triggers positive feedback (Metzler & Scheithauer, 2017; Valkenburg, Peter, & Schouten, 2006). Thus, self-oriented use may promote well-being and protect against increased levels of symptoms, whereas other-oriented social media use, which indicates more frequent exposure to others’ ideal selves (Mascheroni, Vincent, & Jimenez, 2015; Yau & Reich, 2019), may generate upwards social comparisons and thus psychological distress. On the other hand, although negative feedback is found to be rare when adolescents post their own content (Valkenburg et al., 2006), the exposure necessitated by self-oriented social media behavior may put some individuals in a more vulnerable position than does other-oriented behavior, increasing the risk for negative comments (Koutamanis, Vossen, & Valkenburg, 2015) and even cyberbullying, which may forecast depressive symptoms (Pham & Adesman, 2015). As previously noted, posting “selfies” has been associated with poorer body esteem via increased appearance comparison (McLean, Jarman, & Rodgers, 2019), and may thus be a pathway to internalizing symptoms (Choukas-Bradley et al., 2022). In accordance with the opposing assumption that self-oriented social media behavior may both be positively and negatively related to mental health, recent research shows posting (i.e., self-oriented social media use) to be both associated with more internalizing symptoms (Svensson, Johnson, & Olsson, 2022) and more affective well-being (Karsay, Matthes, Schmuck, & Ecklebe, 2022).

### 1.6. Summary and the present study

Research examining the impact of social media use on adolescent mental health has flourished in recent years. According to an umbrella review (with 27 meta-analyses and reviews included), the majority of reviews interpret the evidence as ‘weak’ or ‘inconsistent’ (Valkenburg, Meier, & Beyens, 2022), although some studies report the association to be substantial (Abi-Jaoude, Naylor, & Pignatiello, 2020; Neophytou, Manwell, & Eikelboom, 2021; Twenge, 2020). Valkenburg, Meier, and Beyens (2022) noted that 21 of the 25 reviews concluded that evidence was mainly cross-sectional. Overall, there is a need for (1) longitudinal studies; (2) studies not solely relying on self-report; (3) research capturing types (rather than mere frequency); and (4) inquiries separating between, - from within-person effects. The latter is important because between-person differences in social media use cannot explain why a particular adolescent develops more or less depressive and anxiety symptoms—and vice versa. In the current study we thus ask whether changes within an individual’s social media use (e.g., more self-oriented social media use) predict changes in the same individual’s mental health (e.g., more depressive symptoms), not whether he or she differs from others (i.e., between—person effects). The present work meets the four requirements outlined above, in addition to examining bidirectionality and sex/gender-effects.

Due to the lack of a sound theoretical basis and inconsistency of existing research, we do not offer specific hypotheses, but remain open to the existence and strengths of the relations between self- and other-oriented social media behavior and symptoms of depression, social anxiety, and generalized anxiety, and vice versa. The following research questions are addressed: (1) Do increased self- and other-oriented social media use predict changes in DSM-5 defined symptoms of depressive, social anxiety and generalized anxiety disorders two years later after adjustment for prior changes in these variables and between-person differences in their levels? (2) Are the relations bidirectional? (i.e., do more symptoms predict changes in self- and other-orient social media use?); (3) Are the relations different for boys and girls?

## 2. Materials and methods

The present inquiry is based on data from the Trondheim Early Secure Study (TESS), a longitudinal study of children’s mental health and psychosocial development starting at age 4 years. In 2007/2008, all children born four years earlier in Trondheim, Norway (N = 3,456) were invited to participate in the study. Their parents received an invitation letter together with the Strengths and Difficulties Questionnaire (SDQ) version 4–16) (Goodman, 1997), a mental health screening assessment, which they brought to the child’s 4-year health check at a community health center. At the check up, they were informed about the study by the health care nurse and gave their written consent to participate. Nearly all children attended the check-up (97.2%) and 82.2% of those who were asked to participate consented. To increase variance and thus statistical power, participants with higher scores on the SDQ were oversampled, which is accounted for in the analyses (i.e., weighting back to the population estimates). More specifically, children were allocated to four strata according to their SDQ scores (cut-offs: 0–4, 5–8, 9–11, and 12–40), and the probability of selection increased with increasing SDQ scores (0.37, 0.48, 0.70, and 0.89 in the four strata, respectively). Based on this procedure, 1,250 were selected to participate, and among these, 1007 (79.8%) met at the first assessment at the university clinic. Since then, biennial assessments have been conducted (For details, see (Steinsbekk & Wichstrøm, 2018)). Because social media use was measured from age 10 onwards, the present study captures age 10 (2013/2014, n = 704, 52.1% girls, 47.9% boys, Mage = 10.51, SD = 0.17), 12 (2015/2016, n = 666, 51.7% girls, 48.3% boys, Mage = 12.49, SD = 0.15), 14 (2017/2018,n = 635, 52.9% girls, 47.1% boys, Mage = 14.35, SD = 0.16), and 16 (2019/2020, n = 666, 55.0% girls, 44,9% boys, Mage = 16.98, SD = 0.31).

None of the study variables predicted attrition from age 10 to 12. At age 14, attrition was higher in boys (OR = 1.26 (95% CI, 1.01, 1.57), *p* = .038), in participants who displayed less other-oriented social media behavior (OR = 1.01 (95% CI, 1.00, 1.02), *p* = .008), and fewer symptoms of depression (OR = 0.72 (95% CI, 0.59, 0.87), *p* = .001), but the combined effect of these variables for attrition was small (Cox & Snell = 0.027). At age 16, attrition was only predicted by sex, with boys being more likely to drop out (OR = 1.52 (95% CI, 1.22, 1.89), p ≤ .001). However, due to the many attrition tests run, the risk of false discovery should be considered. An overall test which is not subject to such fallacy, the Little’s MCAR test (Little & Rubin, 2002), indicated that data was indeed missing completely at random (χ^2^ = 429.60, df = 447, *p* = .715). The study is approved by The Regional Committee for Medical and Health Research Ethics, Mid-Norway.

### 2.1. Measures

#### 2.1.1. Social media use

Participants were interviewed about their social media use by trained personnel with at least a bachelor’s degree in a relevant field and substantial experience working with youth.

##### 2.1.1.1. Platforms used.

At ages 10, 12 and 14 years, youth were asked to report which social media platforms they use (e.g., Instagram, Snapchat, Twitter). At age 16, this information was gathered by means of the screen time function of the participants’ phones (i.e., most used apps) and was thus objectively measured.

##### 2.1.1.2. Characteristics of use.

At all timepoints, participants were interviewed about characteristics of their use. Self-oriented use was captured by how often the participants reported posting updates and photos on their own social media page. Other-oriented social media use was measured by how often participants reported that they ‘like’ and ‘comment’ on others posts. At age 16, frequency of selfie-posting was specifically addressed, whereas at earlier timepoints assessments did not distinguish between ‘photos other than selfies’ and ‘selfies’. Therefore, self-oriented social media use constituted 3 behaviors at age 16 (‘written posts’, ‘selfies’, ‘photos other than selfies’), and 2 behaviors at age 10, 12 and 14 years (‘written posts’, ‘photos’). Other-oriented social media use constituted 2 behaviors at all timepoints (‘liking’, ‘commenting’). The interviewer specified for the participant what the different categories of social media behavior captured (e.g., ‘How often do you post photos that are not selfies?’ ‘How often do you write/share something on your social media sites (i.e., not photos)?’).

The interviewer asked the same questions at all time-points, and participants reported frequency of the specific behaviors (e.g., age 12: ‘How often do you post photos?’: 1 = ‘every day’; 2 = ‘2–6 times a week’; 3 = ‘once a week‘; 4 = ‘3 times per month’; 5 = ‘more seldom/never’). To ensure we covered what we expected to be an increase in social media use by age and period, we expanded the scale by applying response categories capturing higher frequencies of the behaviors at older ages. At age 16, a 10-point scale was used (1 = ’seldom or never’; 2 = ‘1–3 times a month’; 3 = ‘1 time a week’; 4 = ‘2–6 times a week’; 5 = ‘1 time a day’; 6 = 2–5 times a day’; 7 = ‘6–15 times a day; 8 = ‘16–30 times a day’; 9 = ‘31–60 times a day’; 10 = ‘More than 60 times a day’). When calculating self- and other-oriented behaviors, we recoded response-categories so that they were aligned across timepoints. Higher numbers indicate higher frequency for all social media behaviors captured.

#### 2.1.2. Symptoms of anxiety and depression

At all timepoints, mental health was measured by semi-structured psychiatric interviews, conducted by trained personnel. Participants and parents were interviewed separately, and a symptom was considered to be present if reported by either child or parent. At ages 10, 12 and 14, we used the Child and Adolescent Psychiatric Assessment (CAPA) (Angold & Costello, 2000), a structured interview assessing symptoms based on the diagnostic criteria of the Diagnostic and Statistical Manual of Mental Disorders (DSM-IV). Note that the criteria for the disorders involved have not changed from DSM-IV to DSM-5. Raters blind to all information of the participants recoded 110 audiotapes at age 10. A symptom count of the number of symptoms of major depressive disorder (ICC = 0.87) was created, as were counts of the number of symptoms of social anxiety (ICC = 0.78) and generalized anxiety disorders (ICC = 0.86), respectively. At age 16, symptoms of depression and anxiety were assessed by the Norwegian version of the Schedule for Affective Disorders and Schizophrenia for School-Aged Children (K-SADS) (Kaufman et al., 1997), which is based on the DSM-5. As in earlier waves, participants and parents were separately interviewed and raters blind to all information about them recoded 114 audiotapes, revealing the following inter-rater reliability: Symptoms of major depressive disorder: ICC = 0.81; Social anxiety: ICC = 0.85; Generalized anxiety: ICC = 0.96).

#### 2.1.3. Sex/gender

Sex was coded based on the participant’s national ID number which is based on sex assigned at birth.

### 2.2. Statistical analyses

**Fig. 1.**
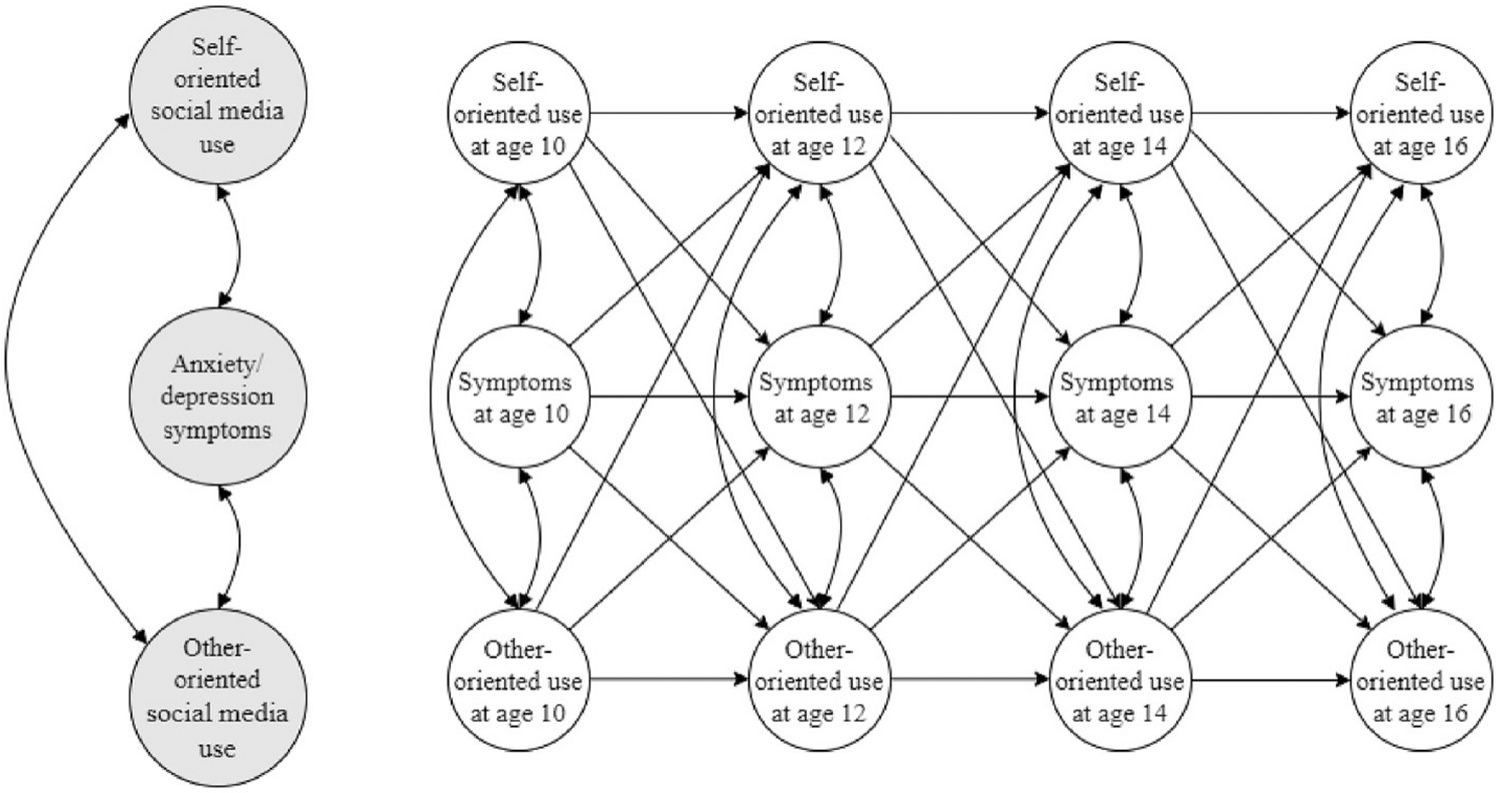
Graphical presentation of the Random Intercept Cross-lagged panel model tested *Note*. The figure displays the relations between random intercept estimates (bigger grey circles) and within-person estimates (smaller white circles) of self-oriented social media behavior, other-oriented social media behavior and symptoms of depression, social anxiety, and generalized anxiety, respectively. One model for symptoms of each of these disorders were tested, thus three in total. Please note that for simplicity reasons, only the latent variables are displayed, not the observed study variables on which they are based.

Analyses were conducted in Mplus version 8.3 (Muthén & Muthén, 1998-2017), using a full information maximum likelihood procedure to handle missing data and probability weights to account for the over-sampling described above (i.e., methods). We estimated Random Intercept Cross-Lagged Panel Models (RI-CLPM) (Hamaker, Kuiper, & Grasman, 2015) to separate within,- from between-person effects, one for each of the three disorders examined. More specifically, latent random intercepts were created for each of the study variables (i.e., capturing the average level of these factors from age 10 to 16). A latent variable was estimated for each of the variables at all time point with the error variance in the observed variable set to 0, thereby transferring the variance to its latent variable. These latent variables thus capture the participant’s deviation from her or his own mean during the observational period. The symptoms of the disorder in question (depression, generalized anxiety, and social anxiety, respectively) and the two types of social media use (self- and other-oriented) at time point t were regressed on the t-1 values of these variables (see Fig. 1). To examine whether the relation between social media behavior and symptoms of depression and anxiety differed between girls and boys, sex-specific models were estimated. Two-sided p-values <.05 were initially regarded as statistically significant. However, due to the large number of tests, we also calculated adjusted p-values to take into account the false discovery rate for p-values <.05 (Benjamini & Hochberg, 1995).

Finally, by means of the same RI-CLPM approach as in the main analyses we conducted sensitivity analyses to test whether the specific behaviors captured by self- and other oriented social media use were differently related to internalizing symptoms. More specifically, rather than using self- and other oriented social media use as the predictors, we tested whether (1) posting updates and photos; and (2) liking and commenting, predicted symptoms of each of the disorders, leaving us with 6 models in total (i.e., 3 disorders x 2 subset of behaviors ((1) and (2) above). Yet again, we accounted for the large number of tests by calculating adjusted p-values (Benjamini & Hochberg, 1995).

## 3. Results

### 3.1. Preliminary results

#### 3.1.1. Social media platforms used

As reported in a former publication (Steinsbekk, Wichstrom, et al., 2021), at ages 10 and 12, Instagram and Snapchat were the most frequently used social media platform for both sexes (Instagram: Age 10: Girls: 42.7%; Boys: 42.5%; Age 12: Girls: 83.2%; Boys: 65.4%; Snapchat: Age 10: Girls: 43.3%; Boys: 34.3%), whereas Facebook topped the list at age 14 (Girls: 93.2%; Boys: 95.7%). At age 16, we registered the three apps the participants used the most based on the screen-time function on their phones. Snapchat was on top of the Number 1 apps list (18.4%), followed by Instagram (17.8%), which applied to both sexes. Details of these data are presented in Supplementary Table S1.

#### 3.1.2. Descriptives of study variables

Means and standard deviations for all study variables for ages 10, 12, 14, and 16 for both sexes are presented in Table 1, whereas Table 2 shows the multivariate correlations between them. Both self - and other-oriented social media behavior increased by age, and particularly so from age 14 to 16 and especially for other-oriented behavior. The sex difference in frequency of these behaviors seems to become less pronounced by age, showing a minor effect at age 16 (Cohen’s *d* = 0.002 and 0.045 for self - and other-oriented use, respectively), whereas being more substantial at earlier ages (Cohen’s d ranging from 0.072 to 0.651). The self - and other oriented social media behaviors were correlated at all timepoints, ranging from small to moderate (*r* = 0.09-0.44). Only a few cross-sectional associations between these social media behaviors and symptoms of depression, social anxiety and generalized anxiety were revealed.

### 3.2. Main results

#### 3.2.1. Self - and other- oriented social media behavior and symptoms of anxiety and depression

Table 3 displays standardized estimates of the RI-CLPM models (Fig. 1) examining the within-person effects of social media use on symptoms of depression, social anxiety, and generalized anxiety and vice versa. All models showed good fit to the data (Depression: CFI = 0.980; TLI = 0.926; RMSEA = 0.037 (90% CI = 0.021, 0.054; *p* = .893); Social anxiety: CFI = 0.979; TLI = 0.922; RMSEA = 0.036 (90% CI = 0.018, 0.052; *p* = .921); Generalized anxiety: CFI = 1.000; TLI = 1.000; RMSEA = 0.000 (90% CI = 0.000, 0.024; *p* = 1.000)). As can be seen, changes in self- and other oriented social media use did not predict changes in participants’ level of symptoms for depression, social anxiety, or generalized anxiety. There were also no significant effects in the opposite direction: changes in depression and anxiety symptoms did not forecast future levels of self- and other oriented social media behavior.

#### 3.2.2. Sex-differences

In accordance with the main findings, sex-specific models of the relations between self - and other-oriented social media behavior and symptoms of depression and generalized anxiety revealed no significant cross-lagged paths in either sex. Please note that due to the large number of tests, we calculated adjusted p-values to take into account the false discovery rate using an online calculator (Benjamini & Hochberg, 1995). Estimates are displayed in Supplementary Tables S2 and S3. Due to the infrequency of social anxiety symptoms, we were unable to estimate-sex specific paths between social media behavior and symptoms of social anxiety.

#### 3.2.3. Sensitivity analyses

Sensitivity analyses examined whether changes in each of the specific behaviors captured by composite self - and other-oriented social media use differently affected future levels of symptoms and vice versa. Here we also calculated adjusted p-values to correct for multiple comparisons (Benjamini & Hochberg, 1995). The results confirmed the null findings of the main analyses: No significant within-person effects from changes in liking, commenting, posting updates and photos to symptoms of anxiety and depression were revealed (Table S4). There were also no significant cross-lagged paths from symptoms of anxiety and depression to changes in liking, commenting, posting updates and photos.

## 4. Discussion

This study examined prospective within-person relations between social media behavior and symptoms of depression, generalized anxiety and social anxiety in a birth-cohort of Norwegian children assessed biennially by means of interviews from age 10–16 years. More specifically, we assessed the potential impact of self-oriented social media behavior (posting updates and photos on one’s own site) and other-oriented social media behavior (liking and commenting on others’ posts) and tested potential bidirectional and sex effects. The results showed that within-person changes (i.e., deviations from one’s own mean level at each time point) in self- and other oriented social media behavior were unrelated to within-person changes in symptoms of depression or anxiety two years later, and vice versa, and this null finding was evident for both boys and girls. Sensitivity analyses examining the specific behaviors captured by self- (i.e., posting updates and photos) and other-oriented (i.e., liking and commenting) behavior, respectively, confirmed the main findings.

### 4.1. Changes in self - and other-oriented social media use is unrelated to future changes in depression and anxiety symptoms

As noted above, reviews conclude that some studies reveal no relations between social media use and mental health in adolescence, and others that social media positively or negatively impacts mental health, and when associations are revealed, they are typically small (Arias-de la Torre et al., 2020; Cunningham et al., 2021; Ivie et al., 2020; McCrae et al., 2017; Odgers & Jensen, 2020; Seabrook et al., 2016; Shin et al., 2022; Valkenburg, Meier, & Beyens, 2022). To the best of our knowledge, the current inquiry is the first to apply a psychiatric interview to assess symptoms of depression and anxiety. In the present study, interviewers probed until they had enough information to code whether a symptom was present or not, according to diagnostic criteria. Thus, as compared to check-lists and other self-report measures, which only modestly correspond with diagnoses established by interviews (Sveen et al., 2013), the interviewer decides whether diagnostic criteria for a symptom are fulfilled, and both parents and adolescents (separately) were interviewed. Hence, not only were the validity of depression and anxiety measures enhanced, but this approach also limits common methods effects in their relation to self-reported social media use.

Given the presumed mechanisms linking social media use and symptoms of depression and anxiety presented above, how come no within-person relations are revealed? As noted, according to the displacement theory (Kraut et al., 1998), increased social media use may decrease face-to-face interactions, potentially impairing mental health. However, a recent review concludes that social media is more likely to replace time spent on other media activities, rather than off-line interaction (Hall & Liu, 2022). In many cases, social media seems to complement rather than displace in-person interactions (Hall & Liu, 2022; Kushlev & Leitao, 2020; Requena & Ayuso, 2019); prospectively predicting more face-to-face interactions (Dienlin, Masur, & Trepte, 2017) and social capital (Hooghe & Oser, 2015). Adolescents even report feeling closer to their friends after using social media (Dredge & Schreurs, 2020; Pouwels, Valkenburg, Beyens, van Driel, & Keijsers, 2021).

Other proposed pathways from social media use to depression and anxiety are cyberbullying (Hamm et al., 2015), co-rumination (Battaglini et al., 2021), negative social comparison (Nesi & Prinstein, 2015), appearance-comparisons (Rapee et al., 2022) and impaired body-image (Choukas-Bradley et al., 2022). Regarding the latter two, research is quite consistent in reporting that being exposed to idealized appearance-related content on social media predicts impaired body image (Vandenbosch et al., 2022), and in a former study of the same sample as applied here, we found that more other-oriented social media use forecasted impaired physical self-esteem in girls (Steinsbekk, Wichstrom, et al., 2021). However, although social media use might be related to such negative outcomes, the present finding does not lend support to the assumption that these are pathways to depression and anxiety symptoms, neither in girls nor boys. The lack of within-person relations further indicate that social media use also do not *protect* against future symptoms of depression and anxiety, although some studies report social media use to be associated with improved self-esteem (Krause, Baum, Baumann, & Krasnova, 2021), sense of belonging, offline social interaction and social capital (Dredge & Schreurs, 2020; Hayes, James, Barn, & Watling, 2022), factors that potentially promote mental health. Though no overall effects were detected, this does not preclude that social media use—and specific social media behaviours or experiences not examined here—might be detrimental to some adolescents. At the same time, some groups may also benefit from social media use. Future research should examine whether subgroups of youth, such as those experiencing bullying, who have lower self-esteem, or who are exposed to specific social media content, are at increased risk. There is indeed reason to believe that some individuals are more susceptible to the negative impacts of social media use than others (Beyens, Pouwels, van Driel, Keijsers, & Valkenburg, 2020, 2021), and such person-specific effects need to be revealed in order to inform guidelines and interventions aimed to promote healthy social media use and mental health.

In the present inquiry, we capture 2-year lags, and we were hence not positioned to detect shorter-term effects which may appear and then vanish between assessment points. However, although one study capturing yearly lags found increased time spent on social media to predict a small increase in self-reported depression (Boers, Afzali, Newton, & Conrod, 2019), the majority of studies examining yearly lags within-person relations have not (Beeres, Andersson, Vossen, & Galanti, 2021; Coyne, Rogers, Zurcher, Stockdale, & Booth, 2020; Heffer, Good, Daly, MacDonell, & Willoughby, 2019; Puukko, Hietajarvi, Maksniemi, Alho, & Salmela-Aro, 2020). Such null findings have also been reported in adolescent studies using even shorter time spans (Jensen et al., 2019; Orben & Przybylski, 2019). Thus, available research does not support the view that prospective relations were lost due to the 2-year follow-ups.

### 4.2. Increased symptoms of depression and anxiety are unrelated to changes in social media use

Our results showed that changes in depression and anxiety did not predict future changes in self- and other oriented social media use. Prior research examining the direction of effects from mental health to social media use is scarce and inconsistent (Sarmiento et al., 2020), but three recent studies have examined within-person effects of changes in adolescents’ self-reported mental health on social media use. Two of these report no such relations (Beeres et al., 2021; Schemer, Masur, Geiss, Muller, & Schafer, 2021), whereas Puukko et al. (2020) found depressive symptoms to increase active social media use. In an adult sample, and using problematic social media use as an outcome, Di Blasi et al. (2022) et al. also found no within-person relations with changes in depression and anxiety symptoms. Our findings extend these results by revealing the same null finding when capturing diagnostically-defined symptoms and examining specific social media behavior. Nevertheless, although individuals who show more symptoms of depression and anxiety over time do not change their social media behavior, their *experiences* with social media use may change.

### 4.3. Limitations

Notable strengths of the current study include the longitudinal design, assessment of diagnostically-defined symptoms of anxiety and depression through clinical interviews of both parents and adolescents, analysing within-person net of between-person effects, and using interviews to capture social media behavior. Even so, we relied on participants to recall and report their frequency of different social media behaviors (i.e., liking, commenting, posting), and such self-reported data is only moderately correlated with logged measurements of use (Parry et al., 2021). Future studies should aim to use objective measures of social media use, such as the Apple and Android screen time function, or social media data captured via “data donations” (van Driel et al.,2022). It should be acknowledged though, that this technology is limited in terms of capturing different aspects of social media behavior such as those assessed here and may nevertheless have to be complemented by self-reported data or technologies that assesses more specific social media behaviors.

Further, studying a phenomenon over time that so rapidly change as social media does, comes with challenges. The first data wave of this study was collected in 2013/2014, and because the frequency of use, the involved apps, and their presentation mode and content have changed since then, we cannot know for sure that findings would be the same if conducted at the present time. At the same time, the basic features of the platforms are the same, at least those assessed here (i.e., liking, commenting, posting), thus there is no immediate reason to believe that findings would not be replicated across time. Whether findings will be replicated in other cultures and countries will be an important area for further research. The prevalence of psychiatric disorders in Norway is low compared to other countries, such as the US (Boe, Heiervang, Stormark, Lundervold, & Hysing, 2021; Wichstrøm et al., 2012), and although the sample is representative of the Norwegian population (Statistics Norway, 2012, 2017), generalization to Non-White, non-Western and marginalized populations should be made with caution. Using data from a clinical sample of adolescents with depression, one study reported that the impact of social media use on adolescents’ experienced affect differed according to ethnicity and race (Nereim, Bickham, & Rich, 2022). Although such findings cannot be generalized to non-clinical populations, future studies should aim to replicate the present results in different cultural contexts.

### 4.4. Conclusions

Although some studies have separated between from within-person effects when examining the relation between social media use and mental health in adolescence, prior studies have emphasized overall time spent on social media and used short questionnaires to measure mental health symptoms. When examining different social media behaviours in a birth cohort sample biennially assessed from age 10–16 years, measuring mental health problems with clinical interviews of multiple reporters, our within-person results revealed no prospective relations between social media use and symptoms of anxiety and depressive disorders—in either direction. The role of social media in mental health is complex, and likely influenced by a range of factors, including specific social media behaviours and experiences, as well as individual differences in adolescents’ responses to use. Findings accord with the view that the frequency with which adolescents engage in behaviours like posting, liking, and commenting on others’ posts does not influence their risk for symptoms of depression and anxiety. Further, results highlight the need for future research examining the nuances of *how* and *for whom* social media use results in positive and negative mental health outcomes.

## Supplementary Material

Appendix A. Supplementary data

## Figures and Tables

**Table 1:** Means and SD of all study variables.

	Total (*N* = 810)		Girls (*n* = 424)		Boys (*n* = 386)		Sex differences		
	Mean	*SD*	Mean	*SD*	Mean	*SD*	Δχ2 (Δdf)	*p*	Cohen’s *d*
Self-oriented social media use per week age 10	2.58	6.35	3.01	7.18	2.10	5.23	5.56 (1)	.018	.145
Self-oriented social media use per week 12	4.89	18.07	5.56	19.07	4.27	16.90	17.81 (1)	≤.001	.072
Self-oriented social media use per week 14	4.58	8.00	5.09	9.42	3.94	6.02	5.26 (1)	.002	.146
Self-oriented social media use per week 16	14.19	74.00	14.23	75.53	14.38	71.66	4.11 (1)	.043	.002
Other-oriented social media use per week age 10	4.34	6.64	5.08	7.18	3.53	5.89	6.45 (1)	.011	.236
Other-oriented social media use per week age 12	9.98	9.14	12.64	9.14	7.01	8.12	50.59 (1)	≤.001	.651
Other-oriented social media use per week age 14	15.02	9.01	17.20	8.59	12.55	8.86	27.30	≤.001	.532
Other-oriented social media use per week age 16	131.556	170.65	134.81	164.99	127.09	177.54	9.65 (1)	.002	.045
Symptoms of depression age 10	0.53	0.89	0.60	1.00	0.46	0.75	4.64 (1)	.031	.158
Symptoms of depression age 12	0.63	1.09	0.70	1.08	0.53	1.09	2.32 (1)	.128	.157
Symptoms of depression age 14	0.85	1.38	1.05	1.58	0.61	1.07	17.68 (1)	≤.001	.326
Symptoms of depression age 16	0.26	0.97	0.31	1.03	0.20	0.89	1.76 (1)	.184	.114
Symptoms of social anxiety age 10	0.03	0.21	0.03	0.22	0.03	0.64	.08	.771	.000
Symptoms of social anxiety age 12	0.05	0.26	0.05	0.26	0.05	0.84	.10 (1)	.761	.000
Symptoms of social anxiety age 14	0.09	0.34	0.11	0.39	0.06	0.89	3.62 (1)	.057	.073
Symptoms of social anxiety age 16	0.45	0.93	0.54	1.03	0.32	0.78	9.42 (1)	.002	.241
Symptoms of generalized anxiety age 10	1.04	1.18	1.00	1.18	1.10	1.17	1.76 (1)	.184	.085
Symptoms of generalized anxiety age 12	0.92	1.19	1.00	1.25	0.84	1.11	3.05 (1)	.081	.135
Symptoms of generalized anxiety age 14	1.17	1.39	1.40	1.53	0.90	1.15	21.86 (1)	≤.001	.369
Symptoms of generalized anxiety age 16	0.99	1.99	1.31	2.21	0.59	1.58	17.07 (1)	≤.001	.374

*Note*: Δχ2 = Sattora-Bentler Scaled Chi-Square Difference. Self-oriented social media use captures the frequency of posting updates, photos, and videos on one’s own social media site. Other-oriented social media use captures the frequency of liking and commenting others’ posts. Symptoms of depression and anxiety are assessed by DSM-5 based psychiatric interviews. The table displays number of symptoms, which have the following possible ranges: Depression: 0–9; Social anxiety: 0–2; Generalized anxiety: 0–6.

**Table 2:** Correlations between all study variables.

	1	2	3	4	5	6	7	8	9	10	11	12	13	14	15	16	17	18	19
1. SELF_10_	–																		
2. OTHER_10_	.44***	–																	
3. DEP_10_	−.02	−.02	–																
4. SOC_10_	−.04**	−.07***	.31**	–															
5. GAD_10_	−.02	−.30	.42***	.21***	–														
6. SELF_12_	.05	.04	.05	.03	.02	–													
7. OTHER_12_	.17***	.32***	−.03	.00	−.04	.09***	–												
8. DEP_12_	−.03	.04	.33***	.07	.24***	.03	−.07	–											
9. SOC_12_	−.04*	−.03	.21***	.37***	.23***	−.00	−.02	.23*	–										
10. GAD_12_	−.03	.05	.32***	.14*	.35***	.05	.06	.46***	.29***	–									
11. SELF_14_	.02	.03	.04	−.03	−.02	−.01	.15***	.06	.11	.09	–								
12. OTHER_14_	.11**	.25***	−.03	−.08	−.04	.12***	.48***	−.05	−.04	.05	.27***	–							
13. DEP_14_	−.03	.07	.38***	.13	.18***	.04	.02	.56***	.36***	.35***	.07	.04	–						
14. SOC_14_	−.01	−.02	.21***	.20**	.15**	−.02	−.05	.21***	.37***	.22***	.01	−.03	.36***	–					
15. GAD_14_	−.03	.00	.34***	.15**	.31***	.06	.05	.40***	.24***	.46***	.04	.04	.64***	.44***	–				
16. SELF_16_	.15	.06	−.04	.01	−.02	−.01	.12*	−.06**	−.03	−.05	−.01	.07	−.03	−.03	−.04	–			
17. OTHER_16_	.12	.06	.01	.01	.05	−.01	.15***	−.06	−.02	.05	.08	.20***	−.01	−.01	.04	.13*	–		
18. DEP_16_	−.01	−.06	.23***	.13	.13*	−.01	−.05	.24**	.07	.16**	−.01	−.07	.29***	.07	.14**	−.02	.05	–	
19. SOC_16_	−.06	−.05	.25***	.21**	.12**	−.06***	−.06	.23***	.30***	.16***	.05	−.08	.34***	.37***	.30***	−.02	.01	.29***	–
20. GAD_16_	.01	−.03	.31***	.15*	.20***	−.02	−.03	.30***	.25***	.35***	.10	.01	.31***	.11*	.26***	−.06***	−.01	.31***	.38***

*Note*: *** = *p* ≤ .001; ** = *p* ≤ .01; * = *p* < .05.

**Table 3:** RI-CLPM estimates of the relations between self - and other-oriented social media behavior and symptoms of depression, social anxiety and generalized anxiety respectively (N = 810).

Parameters	Symptoms of depression			Symptoms of social anxiety			Symptoms of generalized anxiety		
	*β*	95% CI	*p*	*β*	95% CI	*p*	*β*	95% CI	*p*
Correlations^1^									
SELF_int_ ↔ SYMPT_int_	−.424	−1.980, 1.132	.509	−.570	−.714, .573	.263	−.067	−.705, .572	.836
OTHER_int_ ↔ SYMPT_int_	−.199	−.453, .054	.117	−.276	−.776, .224	.209	−.083	−.325, .159	.500
SELF_int_ ↔ OTHER_int_	.654	−1.704, 3.011	.646	.643	−.952, 2.238	.498	.788	−.981, 2.557	.440
Within-person effects									
SELF_10_ → SYMPT_12_	−.062	−.198, .075	.371	−.135	−.321, .051	.165	−.110	−.299, .079	.255
SELF_12_ → SYMPT_14_	.118	−.001, .238	.051	.008	−.084, .100	.862	.060	−.069, .190	.363
SELF_14_ → SYMPT_16_	.012	−.131, .156	.867	.076	−.018, .171	.118	.075	−.035, .184	.187
OTHER_10_ → SYMPT_12_	.148	.000, .296	.063	.148	−.024, .320	.107	.164	−.020, .349	.082
OTHER _12_ → SYMPT_14_	.014	−.098, .125	.811	−.054	−.150, .042	.271	.033	−.093, .158	.611
OTHER _14_ → SYMPT_16_	−.063	−.236, .109	.475	−.059	−.181, .063	.338	.012	−.100, .123	.836
SYMPT_10_ → SELF_12_	.090	−.060, .240	.233	.089	−.093, .271	.327	−.011	−.143, .120	.867
SYMPT_12_ → SELF_14_	.007	−.108, .122	.910	.095	−.023, .213	.126	.071	−.055, .197	.275
SYMPT_14_ → SELF_16_	.036	−.072, .144	.516	.056	−.039, .152	.259	.002	−.099, .103	.968
SYMPT_10_ → OTHER_12_	.011	−.103, .125	.849	.049	−.097, .194	.502	−.047	−.036, .216	.432
SYMPT_12_→ OTHER_14_	−.020	−.175, .135	.799	.037	−.127, .202	.659	.090	−.153, .056	.156
SYMPT_14_ → OTHER_16_	−.027	−.134, .079	.614	−.040	−.138, .057	.410	−.049	−.143, .120	.362
SELF_10_ → OTHER_12_	.125	−.022, .258	.096	.116	−.022, .255	.098	.103	−.043, .249	.165
SELF_12_ → OTHER _14_	.050	−.047, .248	.614	.035	−.161, .231	.725	.024	−.180, .229	.815
SELF_14_ → OTHER _16_	.120	−.031, .271	.117	.111	−.137. .259	.139	.108	−.049, .264	.175
OTHER_10_ → SELF_12_	−.146	−.305, .013	.079	−.144	−.307, .019	.089	−.144	−.310, .023	.098
OTHER _12_ → SELF_14_	.112	−.071, .294	.236	.100	−.079, .280	.277	.087	−.106, .280	.379
OTHER _14_ → SELF_16_	.046	−.131, .222	.615	.041	−.132, .213	.645	.018	−.159, .196	.840
Stability effects									
SELF_10_ →SELF_12_	.326	.167, .484	≤.001	.312	.150, .474	≤.001	.306	.139, .473	≤.001
SELF_12_ → SELF_14_	.171	.050, .292	.007	.158	.035, .280	.013	.163	.041, .284	.010
SELF_14_ → SELF_16_	.13	.027, .236	.013	.116	.014, .219	.024	.127	.025, .230	.013
OTHER_10_ → OTHER _12_	.125	−.010, .259	.073	.124	−.012, .260	.077	.124	−.011, .260	.077
OTHERS_12_ → OTHER _14_	.256	.083, .429	.006	.255	.077, 433	.007	.249	.068, .429	.009
OTHER_14_ → OTHER _16_	.262	.090, .435	.002	.261	.088, .434	.002	.261	.088, .435	.003
SYMPT_10_ → SYMPT_12_	.120	−.106, .346	.307	.183	−.292, .657	.468	−.010	−.231, .211	.929
SYMPT_12_ → SYMPT_14_	.452	.276, .628	≤.001	.266	.002, .530	.023	.205	.016, .395	.025
SYMPT_14_ → SYMPT_16_	.125	−.105, .355	.282	.305	.149, .462	≤.001	.098	−.028, .225	.129

*Note*: ^1^Correlations between the intercepts of the study variables (see Fig. 1). SELF=Self-oriented social media behavior; OTHER= Other-oriented social media behavior; SYMPT= Symptoms of depression, social anxiety and generalized anxiety, respectively (according to top row); _int_ = Intercept; _10, 12, 14, 16_ = Participant age at the time of assessment.

## Data Availability

The authors do not have permission to share data.

## Footnotes

1 The term sex/gender is used in the literature review given that prior studies have often conflated these terms. For the Results and the Discussion, however, we use sex, which accords the measure applied in our study.
