# Supplementary material for: Social media behaviors and symptoms of anxiety and depression. A four-wave cohort study from age 10–16 years
Source: Comput Human Behav. Author manuscript; Available in PMC 2024 Oct 29. (PMC11521397; doi:10.1016/j.chb.2023.107859)
Supplement: Appendix A. Supplementary data [file NIHMS1985252-supplement-Appendix_A__Supplementary_data.zip › 1-s2.0-S0747563223002108-mmc3.docx]

| **Parameters** | **Symptoms of generalized anxiety** | | | | | | | |
| --- | --- | --- | --- | --- | --- | --- | --- | --- |
|  | **Boys** (n= 386) | | | | **Girls** (n= 424) | | | |
| Within-person effects | *β* | 95% CI | *p* | *p** | *β* | 95% CI | *p* | *p** |
| SELF_10_ SYMPT_12_ | -.165 | -.416,.087 | .199 | .705 | -.089 | -.393, .215 | .565 | .693 |
| SELF_12_ SYMPT_14_ | .153 | -.100,.405 | .256 | .705 | .011 | -.124,.145 | .876 | .946 |
| SELF_14_ SYMPT_16_ | .093 | -.148,.334 | .459 | .729 | .051 | -.104,.206 | .516 | .667 |
| OTHER_10_ SYMPT_12_ | .363 | .044,.682 | .017 | .250 | .256 | -.093,.604 | .175 | .502 |
| OTHER _12_ SYMPT_14_ | .012 | -.315,.339 | .943 | .943 | .024 | -.129,.177 | .755 | .849 |
| OTHER _14_ SYMPT_16_ | .153 | -.157,.463 | .313 | .705 | .038 | -.125,.202 | .654 | .768 |
| SYMPT_10_ SELF_12_ | -.008 | -.186, .169 | .926 | .943 | -.074 | -.299,.150 | .519 | .667 |
| SYMPT_12_ SELF_14_ | .044 | -.207,.296 | .730 | .896 | .159 | -.057,.375 | .186 | .502 |
| SYMPT_14_ SELF_16_ | .182 | -.054,.418 | .106 | .572 | -.064 | -.198, .071 | .348 | .626 |
| SYMPT_10_ OTHER_12_ | -.066 | -.220, .087 | .418 | .705 | .000 | -.161,.162 | .997 | .997 |
| SYMPT_12_ OTHER_14_ | .417 | .046,.787 | .021 | .250 | .201 | -.159,.561 | .318 | .626 |
| SYMPT_14_ OTHER_16_ | .089 | -.099,.277 | .352 | .705 | -.125 | -.305,.054 | .170 | .502 |
| SELF_10_ OTHER_12_ | .009 | -.225,.243 | .941 | .943 | .113 | -.038,.263 | .145 | .502 |
| SELF_12_ OTHER _14_ | -.020 | -.317,.277 | .894 | .943 | .174 | -.100,.448 | .223 | .547 |
| SELF_14_ OTHER _16_ | .096 | -.118,.309 | .377 | .705 | .203 | -.005, .410 | .052 | .351 |
| OTHER_10_ SELF_12_ | -.197 | -.478, .084 | .174 | .705 | -.099 | -.354,.157 | .452 | .667 |
| OTHER _12_ SELF_14_ | .089 | -.193,.370 | .532 | .756 | .080 | -.104,.264 | .386 | .635 |
| OTHER _14_ SELF_16_ | .025 | -.290, .241 | .856 | .943 | -.097 | -.316,.122 | .400 | .635 |
| Stability effects |  |  |  |  |  |  |  |  |
| SELF_10_ SELF_12_ | .305 | -.008,.618 | .037 | .250 | .304 | .060,.548 | .007 | .189 |
| SELF_12_ SELF_14_ | .097 | -.132, .325 | .414 | .705 | .231 | -.014,.476 | .078 | .421 |
| SELF_14_ SELF_16_ | .069 | -.148,.287 | .532 | .756 | .256 | .035,.476 | .018 | .243 |
| OTHER_10_ OTHER _12_ | .300 | .053, .546 | .031 | .250 | .056 | -.096,.208 | .475 | .667 |
| OTHER_12_ OTHER _14_ | .124 | -.340, .587 | .618 | .834 | -.172 | -.550,.205 | .343 | .626 |
| OTHER_14_ OTHER _16_ | .179 | -.159,.516 | .248 | .705 | .011 | -.273,.295 | .939 | .975 |
| SYMPT_10_ SYMPT_12_ | .174 | -152,.499 | .285 | .705 | -.220 | -.536, .096 | .285 | .626 |
| SYMPT_12_ SYMPT_14_ | .183 | -.222,.588 | .366 | .705 | .317 | -.029,.663 | .046 | .351 |
| SYMPT_14_ SYMPT_16_ | -.047 | -.291,.196 | .707 | .896 | .137 | -.067,.342 | .167 | .502 |

**Table S3.** RI-CLPM estimates of the gender-specific relations between self, - and other-oriented social media behavior and symptoms of generalized anxiety (N=810).

*Note*: *= Two-sided p-values <0.05 were initially regarded as statistically significant. However, due to the large number of tests, we also calculated adjusted p-values to take into account the false discovery rate for p-values <.05 (Benjamini & Hochberg, 1995), which are reported here; SELF=Self-oriented social media behavior; OTHER= Other-oriented social media behavior; SYMPT= Symptoms of generalized anxiety; _int_=Intercept; _10, 12, 14, 16_= Participant age at the time of assessment.
