# Supplementary material for: Social media behaviors and symptoms of anxiety and depression. A four-wave cohort study from age 10–16 years
Source: Comput Human Behav. Author manuscript; Available in PMC 2024 Oct 29. (PMC11521397; doi:10.1016/j.chb.2023.107859)
Supplement: Appendix A. Supplementary data [file NIHMS1985252-supplement-Appendix_A__Supplementary_data.zip › 1-s2.0-S0747563223002108-mmc1.docx]

**Table S1.** Apps used according to participants’ screen time phone application at age 16 (N=637).

|  | **No 1 app** | | **No 2 app** | | **No 3 app**^a^ | |
| --- | --- | --- | --- | --- | --- | --- |
|  | Girls (%) | Boys (%) | Girls (%) | Boys (%) | Girls (%) | Boys (%) |
| Snapchat | 23.9 | 13.3 | 15.3 | 11.5 | 3.8 | 3.8 |
| TikTok | 17.8 | 11.5 | 17.5 | 7.2 | 5.2 | 3.5 |
| Instagram | 3.0 | 2.0 | 9.1 | 7.5 | 23.2 | 12.7 |
| YouTube | 1.3 | 5.5 | 1.4 | 3.6 | 2.7 | 3.5 |
| Facebook | * | * | * | * | 1.1 | * |
| Netflix | * | * | * | * | 1.1 | * |
| Safari | * | * | * | * | 2.2 | 1.6 |

*Note*: No 1 app= the most used app according to their phone’s screen time application; No 2 app= The second most used app; No 3 app= The third most used app; ^a^=based on iPhone users only, as the third most used app is not registered by the Android screentime function; *=less than 1%. Only apps reported to be used by more than 1% of either girls or boys are listed.
