# Supplementary material for: Social media behaviors and symptoms of anxiety and depression. A four-wave cohort study from age 10–16 years
Source: Comput Human Behav. Author manuscript; Available in PMC 2024 Oct 29. (PMC11521397; doi:10.1016/j.chb.2023.107859)
Supplement: Appendix A. Supplementary data [file NIHMS1985252-supplement-Appendix_A__Supplementary_data.zip › 1-s2.0-S0747563223002108-mmc2.docx]

**Table S2.** RI-CLPM estimates of the gender-specific relations between self, - and other-oriented social media behavior and symptoms of depression (N=810).

| **Parameters** | **Symptoms of depression** | | | | | | | |
| --- | --- | --- | --- | --- | --- | --- | --- | --- |
|  | **Boys** (n= 386) | | | | **Girls** (n= 424) | | | |
|  | *β* | 95% CI | *p* | *p** | *β* | 95% CI | *p* | *p** |
| Within-person effects |  |  |  |  |  |  |  |  |
| SELF_10_ SYMPT_12_ | -0.074 | -0.310, 0.162 | .556 | .891 | -0.072 | -0.255, 0.110 | .444 | .683 |
| SELF_12_ SYMPT_14_ | 0. 148 | -0.099, 0.394 | .328 | . 805 | 0.057 | 0.095, 0.208 | .455 | .683 |
| SELF_14_ SYMPT_16_ | -0.095 | -0.891, 0.702 | .826 | .930 | -0.068 | -0.295, 0.159 | .556 | .790 |
| OTHER_10_ SYMPT_12_ | 0.235 | -0.233, 0.702 | .285 | .805 | 0.228 | -0.006, 0.462 | .075 | .338 |
| OTHER _12_ SYMPT_14_ | -0.014 | -0.365, 0.338 | .940 | .940 | 0.036 | -0.100, 0.172 | .605 | .813 |
| OTHER _14_ SYMPT_16_ | 0.786 | -1.410, 2.982 | .444 | .882 | -0.056 | -0.367, 0.255 | .725 | .813 |
| SYMPT_10_ SELF_12_ | -0.179 | -0.450, 0.092 | .076 | .684 | 0.162 | -0.078, 0.403 | .176 | .475 |
| SYMPT_12_ SELF_14_ | -0.032 | -0.245, 0.181 | .766 | .929 | 0.068 | -0.110, 0.245 | .442 | .683 |
| SYMPT_14_ SELF_16_ | 0.283 | -0.133, 0.699 | .253 | .805 | 0.010 | -0.132, 0.152 | .887 | .922 |
| SYMPT_10_ OTHER_12_ | -0.063 | -0.220, 0.094 | .435 | .882 | -0.023 | -0.142, 0.096 | .712 | .816 |
| SYMPT_12_ OTHER_14_ | 0.312 | -0.605, 1.230 | .308 | .805 | 0.076 | -0.263, 0.415 | .660 | .816 |
| SYMPT_14_ OTHER_16_ | 0.220 | -0.468, 0.909 | .468 | .882 | -0.040 | -0.230, 0.149 | .674 | .816 |
| SELF_10_ OTHER_12_ | -0.016 | -0.412, 0.379 | .937 | .940 | 0.121 | -0.027, 0.268 | .110 | .371 |
| SELF_12_ OTHER _14_ | 0.082 | -0.336, 0.499 | .686 | .926 | 0.177 | -0.082, 0.437 | .200 | .491 |
| SELF_14_ OTHER _16_ | 0.142 | -0.361, 0.645 | .561 | .891 | 0.205 | 0.006, 0.404 | .047 | .254 |
| OTHER_10_ SELF_12_ | -0.169 | -0.600, 0.262 | .490 | .882 | -0.163 | -0.389, 0.062 | .173 | .475 |
| OTHER _12_ SELF_14_ | 0.036 | -0.235, 0.307 | .798 | .929 | 0.103 | -0.080, 0.286 | .267 | .601 |
| OTHER _14_ SELF_16_ | -0.241 | -1.073, 0.590 | .657 | .926 | -0.091 | -0.320, 0.139 | .455 | .683 |
| Stability effects |  |  |  |  |  |  |  |  |
| SELF_10_ SELF_12_ | 0.267 | -0.094, 0.627 | .133 | .805 | 0.338 | 0.124, 0.551 | .002 | .018 |
| SELF_12_ SELF_14_ | 0.175 | -0.015, 0.366 | .060 | .684 | 0.228 | 0.076, 0.379 | .003 | .020 |
| SELF_14_ SELF_16_ | 0.197 | -0.113, 0.508 | .216 | .805 | 0.230 | 0.092, 0.368 | .001 | .014 |
| OTHER_10_ OTHER _12_ | 0.357 | -0.294, 1.009 | .235 | .805 | 0.062 | -0.083, 0.206 | .406 | .683 |
| OTHER_12_ OTHER _14_ | 0.122 | -0.428, 0.671 | .728 | .929 | -0.149 | -0.515, 0.217 | .399 | .683 |
| OTHER_14_ OTHER _16_ | -0.094 | -1.602, 1.413 | .908 | .940 | 0.008 | -0.279, 0.295 | .958 | .958 |
| SYMPT_10_ SYMPT_12_ | 0.317 | -0.092, 0.725 | .007 | .189 | 0.040 | -0.243, 0.323 | .781 | .844 |
| SYMPT_12_ SYMPT_14_ | 0.417 | -0.043, 0.876 | .238 | .805 | 0.490 | 0.263, 0.718 | ≤.001 | ≤.001 |
| SYMPT_14_ SYMPT_16_ | -0.572 | -3.758, 2.614 | .682 | .926 | 0.296 | -0.030, 0.622 | .107 | .371 |

*Note*: *= Two-sided p-values <0.05 were initially regarded as statistically significant. However, due to the large number of tests, we also calculated adjusted p-values to take into account the false discovery rate for p-values <.05 (Benjamini & Hochberg, 1995), which are reported here; SELF=Self-oriented social media behavior; OTHER= Other-oriented social media behavior; SYMPT= Symptoms of depression; _int_=Intercept; _10, 12, 14, 16_= Participant age at the time of assessment.
