# Supplementary material for: Social media behaviors and symptoms of anxiety and depression. A four-wave cohort study from age 10–16 years
Source: Comput Human Behav. Author manuscript; Available in PMC 2024 Oct 29. (PMC11521397; doi:10.1016/j.chb.2023.107859)
Supplement: Appendix A. Supplementary data [file NIHMS1985252-supplement-Appendix_A__Supplementary_data.zip › 1-s2.0-S0747563223002108-mmc4.docx]

**Table S4.** RI-CLPM estimates of the relations between the specific social media behaviors and symptoms of depression, social anxiety and generalized anxiety, respectively (N=810).

| **Parameters** | **Symptoms of depression** | | | | **Symptoms of social anxiety** | | | | **Symptoms of generalized anxiety** | | | |
| --- | --- | --- | --- | --- | --- | --- | --- | --- | --- | --- | --- | --- |
|  | *β* | 95% CI | *p* | *p** | *β* | 95% CI | *p* | *p** | *β* | 95% CI | *p* | *p** |
| Within-person effects:  Social media behavior symptoms |  |  |  |  |  |  |  |  |  |  |  |  |
| UPDATES_10_ SYMPT_12_ | - .082 | - .200, .036 | .184 | .519 | .208 | -.122,.539 | .207 | .599 | .030 | -.212,.152 | .746 | .914 |
| UPDATES_12_ SYMPT_14_ | - .003 | - .138, .132 | .964 | .984 | .024 | -.063,.111 | .584 | .809 | .064 | -.075,.203 | .368 | .860 |
| UPDATES_14_ SYMPT_16_ | - .050 | - .168, .068 | .423 | .725 | .010 | -.093,.114 | .844 | .963 | .085 | -.059,.230 | .238 | .612 |
| PHOTO_10_ SYMPT_12_ | .131 | .001, .261 | .074 | .266 | -.140 | -.386,.106 | .260 | .599 | .066 | -.109,.241 | .464 | .900 |
| PHOTO_12_ SYMPT_14_ | .135 | .027, .244 | .016 | .082 | -.021 | -.109,.068 | .643 | .828 | .024 | -.109,.158 | .720 | .914 |
| PHOTO_14_ SYMPT_16_ | .022 | - .097, .141 | .717 | .953 | .046 | -.056,.148 | .377 | .702 | .005 | -.130,.120 | .935 | .973 |
| LIKING_10_ SYMPT_12_ | .107 | - .051, .265 | .202 | .519 | .056 | - .139, .251 | .578 | .809 | .026 | - .094, .127 | .768 | .914 |
| LIKING_12_ SYMPT_14_ | .070 | - .038, .177 | .196 | .519 | - .009 | - .123, .105 | .874 | .963 | .053 | - .048, .126 | .382 | .860 |
| LIKING_14_ SYMPT_16_ | .002 | - .168, .172 | .984 | .984 | .018 | - .092, .128 | .749 | .899 | - .004 | - .186, .174 | .946 | .973 |
| COMMENTING_10_ SYMPT_12_ | .024 | - .150, .198 | .785 | .956 | .008 | - .158, .173 | .928 | .963 | .136 | -.019, .245 | .094 | .338 |
| COMMENTING_12_ SYMPT_14_ | - .002 | - .110, .106 | .967 | .984 | - .035 | - .132, .063 | .493 | .809 | .24 | - .090, .132 | .712 | .914 |
| COMMENTING_14_ SYMPT_16_ | - .079 | - .231, .073 | .318 | .636 | - .059 | - .163, .045 | .266 | .599 | .071 | - .059, .251 | .224 | .612 |
| Within-person effects:  Symptoms social media behavior |  |  |  |  |  |  |  |  |  |  |  |  |
| SYMPT_10_ UPDATES _12_ | .101 | - .064, .266 | .227 | .545 | .092 | -.121,.305 | .390 | .702 | .021 | -.128,.170 | .780 | .914 |
| SYMPT_12_ UPDATES _14_ | .007 | - .096, .111 | .889 | .984 | .167 | .024,.309 | .038 | .195 | .095 | -.047,.237 | .190 | .612 |
| SYMPT_14_ UPDATES _16_ | .018 | - .084, .119 | .734 | .953 | .045 | -.037,.127 | .290 | .608 | .013 | -.110,.084 | .794 | .914 |
| SYMPT_10_ PHOTO _12_ | .079 | - .040, .198 | .189 | .519 | .008 | -.179,.194 | .935 | .963 | -.017 | -.153,.120 | .812 | .914 |
| SYMPT_12_ PHOTO _14_ | .003 | - .097, .102 | .959 | .984 | -.030 | -.134,.074 | .565 | .809 | .037 | -.066,.140 | .485 | .900 |
| SYMPT_14_  PHOTO _16_ | .064 | - .051, .179 | .270 | .608 | .108 | -.013,.229 | .079 | .292 | .017 | -.101,.135 | .777 | .914 |
| SYMPT_10_  LIKING _12_ | .020 | - .098, .137 | .741 | .953 | .050 | - .101, .201 | .501 | .809 | -.024 | -.239, .156 | .683 | .914 |
| SYMPT_12_  LIKING _14_ | - .025 | - .160, .110 | .713 | .953 | .041 | - .096, .178 | .558 | .809 | .029 | - .121, .197 | .642 | .914 |
| SYMPT_14_ LIKING _16_ | - .018 | - .123, .087 | .737 | .953 | - .016 | - .110, .079 | .745 | .899 | - .035 | - .241, .117 | .500 | .900 |
| SYMPT_10_ COMMENTING _12_ | -.026 | - .160, .107 | .702 | .953 | .083 | - .015, .180 | .109 | .357 | - .005 | - .188, .174 | .939 | .973 |
| SYMPT_12_ COMMENTING _14_ | - .008 | - .129, .112 | .890 | .984 | - .053 | - .152, .047 | .261 | .599 | .105 | .010, .291 | .036 | .185 |
| SYMPT_14_ COMMENTING _16_ | - .045 | -.138, .048 | .348 | .659 | .002 | - .079, .083 | .969 | .969 | .021 | - .107, .163 | .683 | .940 |
| Stability effects - social media behavior |  |  |  |  |  |  |  |  |  |  |  |  |
| UPDATES_10_  UPDATES _12_ | .168 | .005, .331 | .051 | .204 | .168 | -.016,.352 | .081 | .292 | .163 | -.002,.328 | .060 | .240 |
| UPDATES _12_  UPDATES _14_ | .087 | -.104, .277 | .378 | .680 | .109 | -.081,.300 | .266 | .599 | .073 | -.118,.264 | .459 | .900 |
| UPDATES _14_ UPDATES _16_ | .285 | .155, .415 | ≤.001 | ≤.001 | .295 | .162,.428 | ≤.001 | ≤.001 | .282 | .150,.414 | ≤.001 | ≤.001 |
| PHOTO_10_ PHOTO _12_ | .021 | - .138, .180 | .797 | .953 | .007 | -.167,.181 | .936 | .962 | .002 | -.169,.172 | .986 | .986 |
| PHOTO_12_ PHOTO _14_ | .291 | .147, .435 | ≤.001 | ≤.001 | .280 | .137,.422 | ≤.001 | ≤.001 | .284 | .139,.428 | ≤.001 | ≤.001 |
| PHOTO_14_ PHOTO _16_ | .030 | - .082, .142 | .599 | .014 | .025 | -.082,.133 | .644 | .828 | .024 | -.085,.133 | .666 | .914 |
| LIKING_10_ LIKING _12_ | .196 | .074, .318 | .002 | ≤.001 | .198 | .076, .320 | .002 | .014 | .194 | .077, .349 | .002 | .014 |
| LIKING_12_ LIKING _14_ | .240 | .027, .453 | .032 | .144 | .225 | .011, .440 | .046 | .207 | .233 | .001, .352 | .048 | .216 |
| LIKING_14_ LIKING_16_ | .286 | .125, .446 | ≤.001 | ≤.001 | .279 | .120, .438 | ≤.001 | ≤.001 | .282 | .210, .733 | ≤.001 | ≤.001 |
| COMMENTING_10_ COMMENTING _12_ | .082 | - .071, .236 | .293 | .620 | .080 | - .073, .234 | .304 | .608 | .092 | - .071, .288 | .236 | .612 |
| COMMENTING_12_ COMMENTING _14_ | .206 | .049, .364 | .010 | .060 | .199 | .044, .355 | .012 | .072 | .216 | .061, .374 | .007 | .042 |
| COMMENTING_14_ COMMENTING _16_ | .381 | .259, .502 | ≤.001 | ≤.001 | .373 | .249, .498 | ≤.001 | ≤.001 | .383 | .297, .596 | ≤.001 | ≤.001 |

*Note*: Results of 6 models are displayed (i.e., 3 disorders x 2 subset of behaviors (posting updates + photos; liking + commenting). Within-person relations between the different social media behaviors (e.g., UPDATES_10_ PHOTO_12_) were also estimated, but are not shown. Stability of symptoms is displayed in the main model (Table 3).

*= Two-sided p-values <0.05 were initially regarded as statistically significant. However, due to the large number of tests, we also calculated adjusted p-values to take into account the false discovery rate for p-values <.05 (Benjamini & Hochberg, 1995), which are reported here. UPDATES=posting updates (text, not photos/videos); PHOTO= posting photos/videos; LIKING= liking other’s posts; COMMENTING=commenting on others’ posts; SYMPT= Symptoms of depression, social anxiety and generalized anxiety; _10, 12, 14, 16_= Participant age at the time of assessment.
